# ALDH2 Mediated Ferroptosis Regulation in Ischemia–Reperfusion Injury

**DOI:** 10.1111/jcmm.71072

**Published:** 2026-03-25

**Authors:** Liang Han, Wen Zhai

**Affiliations:** ^1^ Center for Rehabilitation Medicine, Department of Anesthesiology Zhejiang Provincial People's Hospital (Affiliated People's Hospital, Hangzhou Medical College) Hangzhou China; ^2^ Research Institute of Anesthesiology and Perioperative Medicine Hangzhou Medical College Zhejiang Hangzhou China

**Keywords:** ALDH2, ferroptosis, ischemia–reperfusion injury, regulated cell death

## Abstract

Ischemia–reperfusion injury (IRI) is a common complication in diverse clinical settings, including myocardial infarction, stroke, organ transplantation and major surgery. Its pathological core lies in the metabolic disruption caused by blood flow cessation, followed by oxidative stress, inflammatory cascades and cell death upon reperfusion. Aldehyde dehydrogenase 2 (ALDH2), a mitochondrial enzyme best known for its role in aldehyde detoxification, has emerged as an important endogenous protective factor in IRI. ALDH2 limits the accumulation of toxic lipid peroxidation products, preserves mitochondrial function and attenuates oxidative and inflammatory damage in multiple organs. In parallel, ferroptosis, an iron‐dependent form of regulated cell death driven by lipid peroxidation, has been increasingly recognised as a key execution pathway in IRI. Increasing evidence suggests a close intersection between ALDH2 and ferroptosis. This review aims to systematically summarise the interplay between ALDH2 and ferroptosis in IRI, further explore their involvement in other forms of regulated cell death and highlight their points of convergence. Collectively, available data suggest that targeting ALDH2 may represent a promising strategy for limiting ferroptosis and reducing tissue damage in IRI.

## 1 Introduction

Ischemia–reperfusion injury (IRI) is a common and severe pathological process in myocardial infarction, stroke, organ transplantation and major surgical procedures. Its arises from the metabolic disturbances caused by interrupted blood supply during ischemia [1]. The pathophysiology of IRI generally proceeds through distinct stages: (1) Ischemic phase: Ischemia is characterised by insufficient oxygen and nutrient delivery, leading to ATP depletion, metabolic acidosis and the accumulation of toxic metabolites. These changes compromise cellular integrity and viability. (2) Reperfusion phase: Reperfusion, although essential for tissue survival, introduces a sudden influx of oxygen that triggers excessive reactive oxygen species (ROS) production, calcium overload and inflammatory activation, thereby amplifying tissue injury rather than reversing it [2]. Together, ischemia and reperfusion constitute a tightly linked pathological continuum that underlies organ dysfunction. At present, effective therapeutic strategies to prevent the progression of IRI are lacking, making it essential to unravel its molecular mechanisms in order to design novel interventions.

In recent years, studies have increasingly revealed the pivotal role of aldehyde dehydrogenase 2 (ALDH2) in combating IRI. ALDH2 is one of the 19 members of the human ALDH family, predominantly localised in liver mitochondria, where it functions as a tetramer [3]. As a key mitochondrial aldehyde‐metabolising enzyme, ALDH2 has traditionally been considered essential for detoxifying acetaldehyde generated during alcohol metabolism. Exogenous ethanol is metabolised in the liver by alcohol dehydrogenase, the microsomal ethanol oxidation system and cytochrome P450 2E1, producing acetaldehyde. Circulating acetaldehyde exerts various toxic effects. ALDH2 catalyses the removal of two hydrogen atoms from acetaldehyde, converting it into non‐toxic acetate, which is ultimately metabolised into carbon dioxide and water [3, 4]. However, mounting evidence indicates that beyond its role in detoxifying exogenous aldehydes, ALDH2 also eliminates endogenous aldehydes generated under oxidative stress, such as 4‐hydroxynonenal (4‐HNE), malondialdehyde (MDA) and acrolein [5, 6]. Moreover, ALDH2 helps preserve mitochondrial membrane integrity, suppress toxic intracellular pathways and limit tissue and organ injury [7, 8]. Recent studies further highlight the broader relevance of ALDH2, extending beyond its traditional associations with cardiovascular disease, diabetes and neurodegenerative disorders. ALDH2 is now recognised as a critical defender against IRI across multiple organs, including the heart, brain, intestine and kidney [9]. Recent studies on the role of ALDH2 in ischemia–reperfusion injury were presented in Table 1.

**TABLE 1:** Studies involved in the role of ALDH2 in ischemia–reperfusion injury in recent 5 years.

Organ	Cells	Animal model	Mechanism	Year	Function
Heart	Not reported	Mouse hypertrophic preconditioning model	Activate AMPK pathway	2021	Improve mitochondrial energy metabolism [10]
	H9C2 cells	Rat diabetic myocardial I/R model	Inhibit mitoPTP opening/Activate PI3K/AKT/mTOR pathway	2023	Regulate mitochondrial fusion and fission/maintain mitochondrial stability/Reduce mitochondrial ROS level and apoptotic protein expression [11].
	Not reported	OGD/R model	Inhibit ERK/p38 signalling pathway	2023	Inhibite apoptosis/suppress 4‐HNE and MDA of cardiomyocytes [12].
	Not reported	Mouse ALDH2 knockout myocardial I/R model	Activate ALDH2/SIRT3/HIF1α signalling pathway	2024	RIC increase ALDH2 protein levels/inhibit autophagy [13].
	H9c2 cell	Mouse myocardial I/R injury model	Increase mitochondrial potential‐mediated fusion	2021	Increases mitochondrial potential‐mediated fusion/promote the oxygen consumption rate and the function of cardiomyocytes [14].
	CEC	Mouse ALDH2 2*2 E487K knock‐in model	Alleviate 4‐HNE‐mediated CEC injury	2021	Reduce myocardial infarct size and dysfunction and coronary perfusion pressure/increase CEC population and coronary arteriole opening [15].
	Not reported	Isolated heart I/R model	Transcriptionally upregulate ALDH2	2021	Enhance citrate synthase activity/benefit mitochondrial function and energy production [16].
Brain	PC12 cell	Rat cerebral IRI model	Increasing ALDH2 expression/Enhancing ALDH2 activity	2022	Improve the injury of neurons induced by hypoxia/reoxygenation [17].
Brain	HT22 cell/mouse cortical neurons	Not reported	Promoter region hypermethylation of ALDH2	2023	Attenuate OGD/R‐induced cell apoptosis, pyroptosis, ferroptosis and autophag [18].
	Not reported	Swine CA/CPR model	Inhibit NLRP3 inflammasome activation and pyroptosis	2022	Improve cardiac and neurological dysfunction/reduce cardiac and cerebral injuries [19].
	bEnd.3 cells	MCAO/R model	Suppress IL‐1β and IL‐18, Inhibit NLRP3 expression	2025	Enhance bEnd.3 cells viability/Reduce infarct area and neurological impairment [20].
Kidney	HK‐2 cells	Mouse ALDH2−/− kidney IRI model	Activate IκBα/NF‐κB pathway	2023	ALDH2 deficiency promote IκBα/NF‐κB p65 phosphorylation, increase inflammatory factors [21].
	murine RTEC	Mouse AKI models	Activate Beclin‐1 pathway	2021	Enhanced Beclin‐1 phosphorylation, remove ROS via Beclin‐1–induced autophagy [22].
	Not reported	Swine CA/CPR model	Inhibit cell apoptosis and ferroptosis	2022	Alleviate post‐resuscitation renal and intestinal injury [23].
	Not reported	ALDH2 knock‐out C57BL/6 mice	Interacts with NCOR1 to repress Cyp4a transcription	2025	Disrupte lipid metabolism/increas YP4A and 20‐HETE level/Exacerbated renal inflammation/trigger endoplasmic reticulum stress [24].
Lung	Not reported	Swine CA/CPR model	Restore ALDH2 activity	2022	Inhibit cell apoptosis and ferroptosis/alleviate lung injury [25].
Skin	Not reported	Rat McFarlane flap models	Downregulate PINK1/Parkin signalling pathway	2023	Enhance random skin flap viability via inhibiting PINK1/Parkin‐dependent mitophagy, anti‐inflammatory and angiogenesis [26].

Abbreviations: CEC, coronary endothelial cell; HMP, hypothermic machine perfusion; MCAO/R, cerebral artery occlusion/reperfusion; OGD/R, oxygen–glucose deprivation reperfusion; RIC, Remote ischemic conditioning; RTEC, renal tubular epithelial cell; TAC, transverse aortic constriction.

Meanwhile, ferroptosis—a newly characterised form of iron‐dependent regulated cell death (RCD)—is typified by intracellular iron accumulation and consequent oxidative stress, including glutathione peroxidase 4 (GPX4) inactivation, lipid peroxidation and disruption of iron homeostasis and has emerged as a central execution pathway in IRI [27]. Recent evidence shows that ferroptosis not only exacerbates cellular injury during IRI but also amplifies tissue damage by intersecting with inflammatory signalling, apoptosis and necroptosis, thereby representing a critical therapeutic target [28, 29]. Although ferroptosis in IRI is multifaceted, its central role in the injury cascade has been firmly established [30]. Accordingly, targeting ferroptosis has become a promising strategy for mitigating IRI.

**FIGURE 1:**
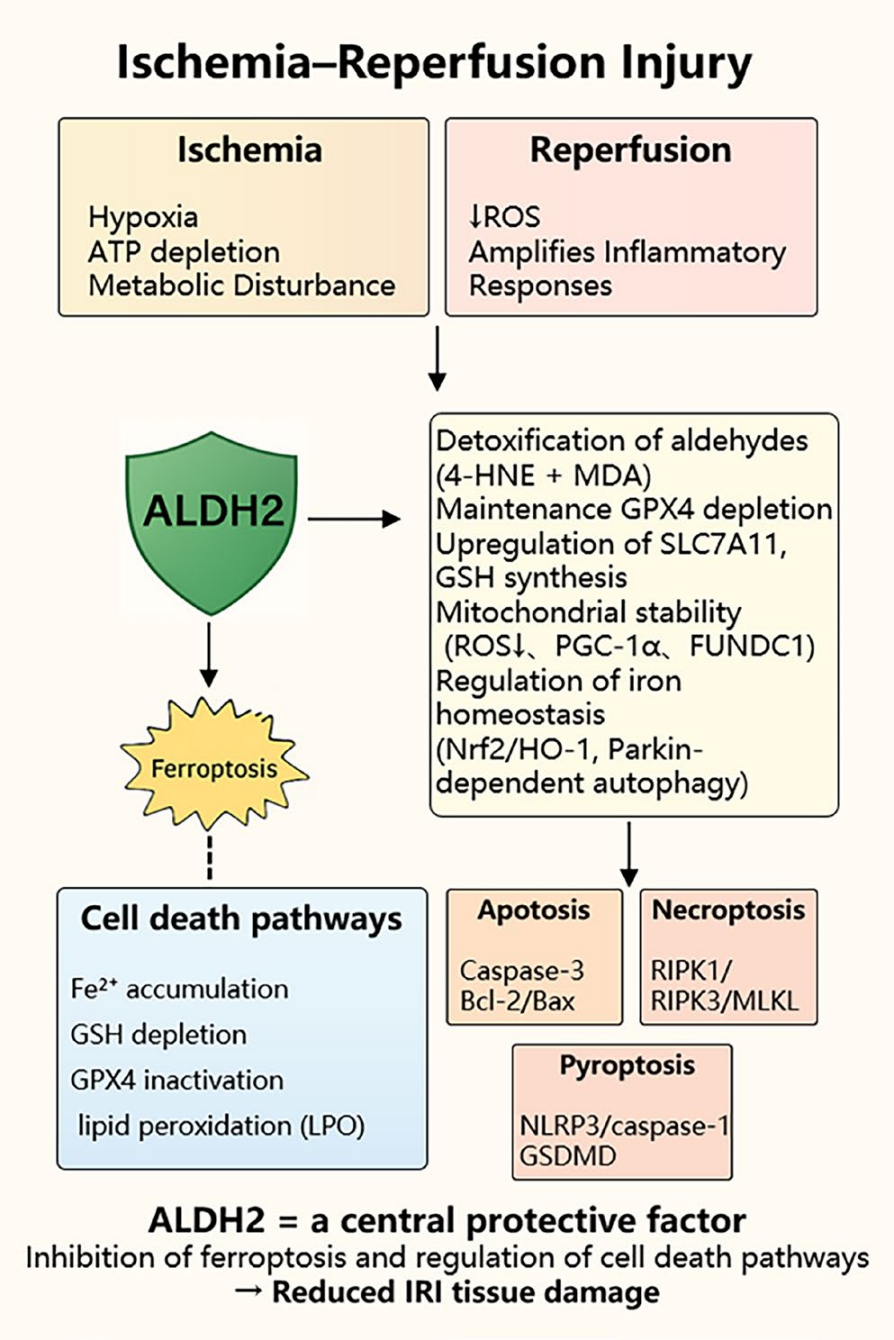
ALDH2 mediated Ferroptosis regulation in ischemia–reperfusion injury. Ischemia–reperfusion leads to multiple forms of regulated cell death, including ferroptosis. ALDH2, represented as a protective shield that protects tissues from IRI‐induced injury.

Notably, the mechanisms of ALDH2 and ferroptosis overlap substantially in IRI: ALDH2 stabilises intracellular homeostasis by detoxifying aldehydes and attenuating lipid peroxidation, which are the central pathological features of ferroptosis. Recent animal studies indicate that alterations in ALDH2 activity significantly affect ferroptosis markers such as GPX4, ACSL4 and 4‐HNE, thereby determining the extent of IRI [31]. Thus, ALDH2 is likely a crucial upstream regulator of ferroptosis in IRI. Based on this, the present review systematically summarises the protective role of ALDH2 in IRI, with emphasis on its interplay with ferroptosis and downstream molecular pathways. We further discuss the convergence of ALDH2 with other forms of programmed cell death. By synthesising recent advances, we aim to provide new mechanistic insights and therapeutic perspectives for targeting IRI (Figure 1).

## 2 Ferroptosis in Ischemia–Reperfusion Injury

**FIGURE 2:**
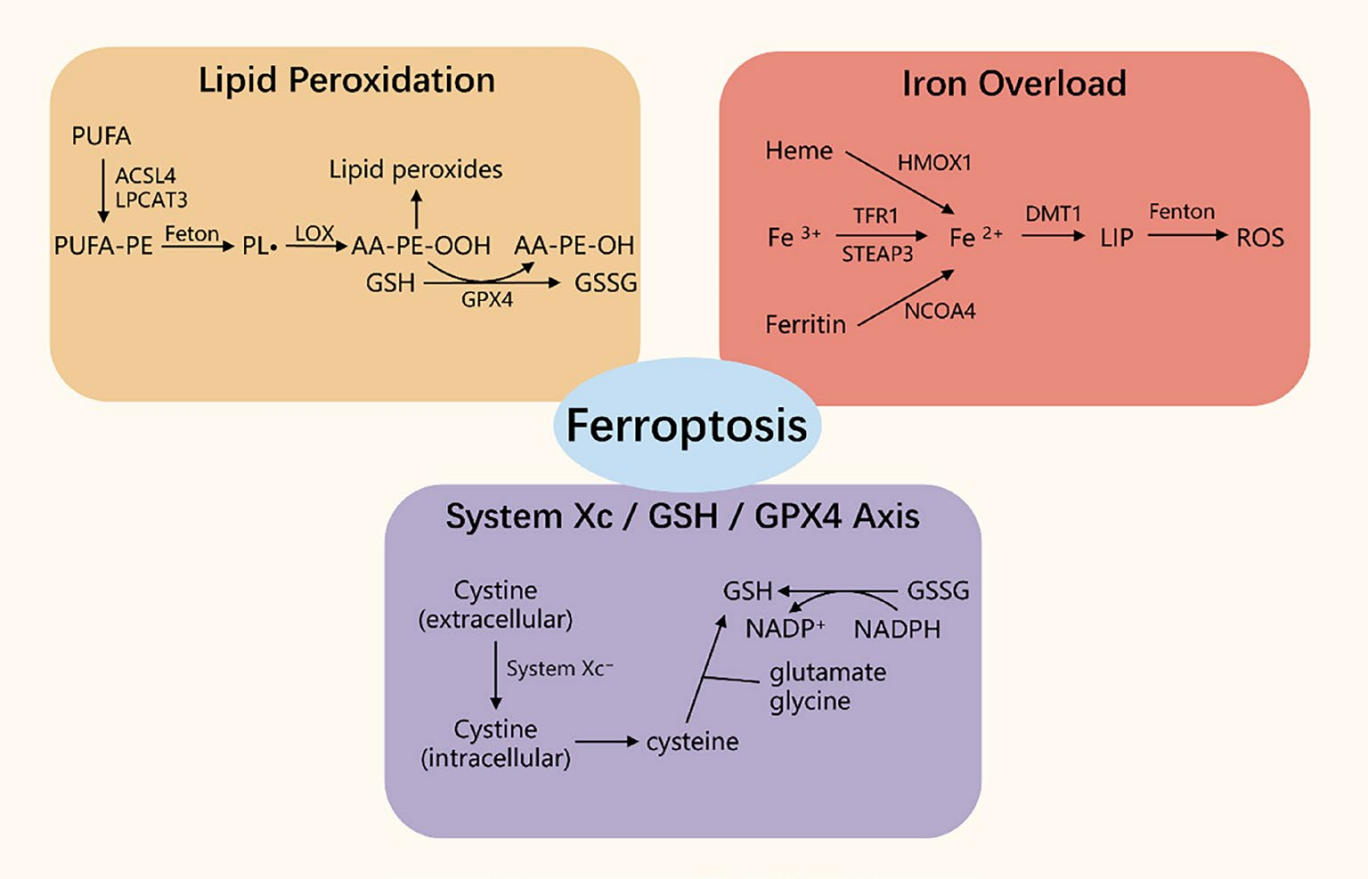
Three major mechanisms of Ferroptosis triggered by IRI: lipid peroxidation: polyunsaturated fatty acids (PUFA), esterified into membrane phosphatidylethanolamines (PE) via ACSL4 and LPCAT3, become the most susceptible targets for peroxidation (PUFA‐PE). PUFA‐PE generates lipid radicals (PL•) through the Fenton reaction, which are then converted into lipid hydroperoxides (PL‐OOH) under the action of lipoxygenases (LOXs). Glutathione peroxidase 4 (GPX4), using glutathione (GSH) as a reducing agent, reduces toxic PL‐OOH to PL‐OH. Iron Overload: Transferrin (Tf) bound to iron (Fe^3+^) is internalised via transferrin receptor 1 (TfR1) and STEAP3 reduces Fe^3+^ to Fe^2+^. Ferritin releases Fe^2+^ into the cytoplasm through NCOA4‐mediated ferritinophagy. Heme is degraded by heme oxygenase 1 (HMOX1). Frataxin imports iron into mitochondria via Fe–S cluster assembly. Hepcidin inhibits iron export through ferroportin 1 (FPN1), leading to Fe^2+^ accumulation in the labile iron pool (LIP), which then participates in Fenton reactions. Imbalance of the System Xc^−^/GSH/GPX4 Axis: The Xc^−^ system imports cystine into the cells. Cystine is reduced to cysteine, which then combines with glycine and glutamate to synthesise glutathione (GSH). Under the catalysis of GPX4, hydrogen peroxide and lipid peroxides are reduced, while GSH is oxidised to oxidised glutathione (GSSG).

Ferroptosis is a newly recognised form of RCD, characterised by intracellular iron accumulation, inactivation of GPX4, depletion of GSH and consequent massive accumulation of lipid peroxides [27]. Unlike apoptosis, necroptosis, or pyroptosis, the core execution of ferroptosis involves lipid peroxidation and disruption of iron homeostasis. Evidence indicates that ferroptosis predominantly occurs during the reperfusion phase rather than the ischemic phase of IRI [32]. Previous studies have confirmed that ferroptosis is a significant mode of cell death in multiple organs including the heart, brain, kidneys and liver [1]. Mechanistically, IRI‐induced ferroptosis mainly involves pathways related to lipid metabolism, iron metabolism and amino acid metabolism (Figure 2) [33].

### 2.1 Lipid Peroxidation

Lipid metabolism plays a pivotal role in ferroptosis during IRI. Lipid peroxidation in ferroptosis originates from polyunsaturated fatty acids (PUFAs), particularly the peroxidation of arachidonic acid‐phosphatidylethanolamine (AA‐PE) [34]. PUFAs have a high affinity for reactive oxygen species (ROS) and during reperfusion, ROS exacerbate PUFAs oxidation via the Fenton reaction. ROS such as hydroxyl radicals (OH•) and hydrogen peroxide (H_2_O_2_) abstract hydrogen from PUFAs, generating lipid ROS (L^−^), which are further oxidised to lipid peroxyl radicals (LOO^−^) [35]. Ferroptosis is triggered when LOO• levels exceed the capacity of cellular antioxidant systems [36]. In mice, ischemia‐induced ALOX‐15 oxidises AA‐PE to oxidised phosphatidylethanolamines, initiating lipid peroxidation during reperfusion and leading to ferroptosis [32]. Lipoxygenases (LOXs) play a central role in this process, catalysing ROS production and the oxidation of AA‐PE to AA‐OOH‐PE [1, 37]. Studies in a murine renal IRI model show that LOX expression increases progressively with reperfusion time, peaking at 5 h post‐reperfusion and LOX inhibitors can mitigate IRI‐induced damage [38]. Similarly, various toxic aldehydes produced during lipid peroxidation should not be ignored either. Notably, 4‐HNE and MDA have emerged as markers of ferroptosis and amplify lipid peroxidation in a positive feedback loop, further damaging mitochondria and the antioxidant system. 4‐HNE has been shown to inhibit intracellular accumulation of aldehyde dehydrogenase 1B1 through activation of EIF4E. Supra‐physiological levels of 4‐HNE also activate NADPH oxidase 1 (NOX1), thereby promoting ferroptosis [39]. Additionally, indicators such as the GSH/oxidised glutathione (GSSG) ratio, Fe^2+^ accumulation and ROS levels are widely used to monitor ferroptosis in cells [40, 41].

### 2.2 Iron Overload

Disruption of iron homeostasis is another central factor in ferroptosis. Iron promotes ROS accumulation via the Fenton reaction and serves as an essential cofactor for enzymes such as LOXs [42]. Studies have shown that the Fenton reaction leads to excessive OH• accumulation, enhancing lipid peroxidation, generating 4‐hydroxynonenal (4‐HNE) and activating cyclooxygenase‐2 (COX2). COX2, a pro‐inflammatory enzyme, can further promote intracellular inflammation and influence neighbouring cells via vesicular release [43, 44]. Furthermore, 4‐HNE forms adducts with mitochondrial proteins, causing mitochondrial dysfunction, increasing ROS production and ultimately activating nuclear factor erythroid 2–related factor 2 (NRF2)‐mediated antioxidant responses [45] Iron overload plays a critical role in the initiation and progression of ferroptosis through several mechanisms: (1) Heme oxygenase‐1 (HO‐1) mediates haemoglobin degradation, releasing Fe^2+^ and inducing iron overload [46]; (2) Ferritinophagy: Nuclear receptor coactivator 4 (NCOA4)‐mediated autophagic degradation releases Fe^2+^ from ferritin in autophagosomes [47]; (3) Transferrin receptor 1 (TfR1) facilitates cellular iron uptake from transferrin via endocytosis, inducing ferroptosis during IRI, a process upregulated by p53 through deubiquitination by ubiquitin‐specific protease 7 (USP7) [48]. Ultimately, iron accumulated via multiple pathways is released into the labile iron pool (LIP) in the cytoplasm, driving ROS generation and lipid peroxidation. Studies have shown that SUMO‐specific protease 2 (SENP2) alleviates ferritinophagy‐dependent ferroptosis by catalysing the deSUMOylation of NCOA4, thereby protecting the myocardium and promoting functional recovery after myocardial ischemia–reperfusion injury [49]. Inhibitors targeting ferritinophagy can significantly mitigate organ damage and DNA methyltransferase 1 (DNMT‐1) inhibitors can reduce ferroptosis in diabetic myocardial IRI by suppressing NCOA4‐mediated ferritinophagy, alleviating myocardial injury [50]. Targeting transferrin receptor 1 (TfR1) also counteracts abnormal iron accumulation and ferroptosis. HUWE1 has been reported to regulate iron metabolism by promoting ubiquitination‐mediated degradation of TfR1, thereby inhibiting ferroptosis and ameliorating acute liver injury, suggesting potential protective effects [51]. During ferroptosis, ferritin interacts with transferrin receptors to facilitate iron endocytosis, increasing lysosomal Fe^2+^ levels, which further promotes ROS generation and lipid peroxidation, contributing to ferroptotic cell death [52].

### 2.3 Imbalance of the System Xc^−^/GSH/GPX4 Axis

The system Xc^−^/GSH/GPX4 axis is a primary pathway inhibiting ferroptosis. System Xc^−^, composed of SLC7A11 and SLC3A2, mediates the import of cystine into the cell. Within the cytoplasm, cystine is reduced to cysteine, which then combines with glycine and glutamate to synthesise GSH. Under the catalytic action of GPX4, H_2_O_2_ and LPO are reduced while GSH is oxidised to GSSG [53]. During IRI, downregulation of SLC7A11 suppresses system Xc^−^, resulting in decreased GPX4 activity and GSH depletion, causing accumulation of toxic lipid ROS and triggering ferroptosis [54]. Studies have shown that in myocardial IRI models, GPX4 overexpression significantly reduces lipid peroxidation and cell death in cardiomyocytes, thereby protecting cardiac function. Conversely, GPX4‐deficient mice exhibit more severe myocardial injury, elevated lipid peroxidation and impaired cardiac function following I/R injury, highlighting the protective role of GPX4 in myocardial IRI [55]. Selenium is essential for maintaining GPX4 activity; pharmacological Se supplementation can upregulate GPX4 and other selenoproteins via activation of transcription factors TFAP2c and Sp1, protecting neurons from ferroptosis and other forms of cell death and improving neurological outcomes in models of cerebral haemorrhage or ischemia [56]. Intra‐articular injection of Ferrostatin‐1 reduces ROS and MDA generation in chondrocytes, alleviates inhibition of GPX4 and SLC7A11, thereby suppressing ferroptosis, decreasing MMP13 levels, maintaining type II collagen and slowing osteoarthritis progression [57]. Additionally, 4‐HNE can carbonylate cysteine 93 on GPX4 and cysteine 247 on the deubiquitinase OTUD5, disrupting their interaction, promoting K48‐linked polyubiquitination and degradation of GPX4 and directly inducing ferroptosis in cardiomyocytes [40].

In summary, ferroptosis represents a critical pathological process in IRI, encompassing lipid peroxidation, iron homeostasis disruption and exhaustion of antioxidant systems. Ferroptosis is also highly coupled with the inflammatory response in IRI. Reactive aldehydes generated during lipid peroxidation, such as 4‐HNE, can activate NOX1 and COX2, promoting the release of pro‐inflammatory cytokines and further amplifying cellular damage [58]. Therefore, ferroptosis not only acts as a direct executor of IRI but also amplifies tissue damage through inflammatory and oxidative stress loops. Accumulating experimental evidence indicates that interventions targeting ferroptosis (such as the iron chelator deferoxamine, the ferroptosis inhibitor ferrostatin‐1, or GPX4 activators) can effectively mitigate tissue injury in IRI [29] Consequently, a deeper understanding of the molecular mechanisms of ferroptosis not only elucidates the pathological essence of IRI but also provides novel potential targets for clinical intervention.

## 3 ALDH2 and Ferroptosis

**FIGURE 3:**
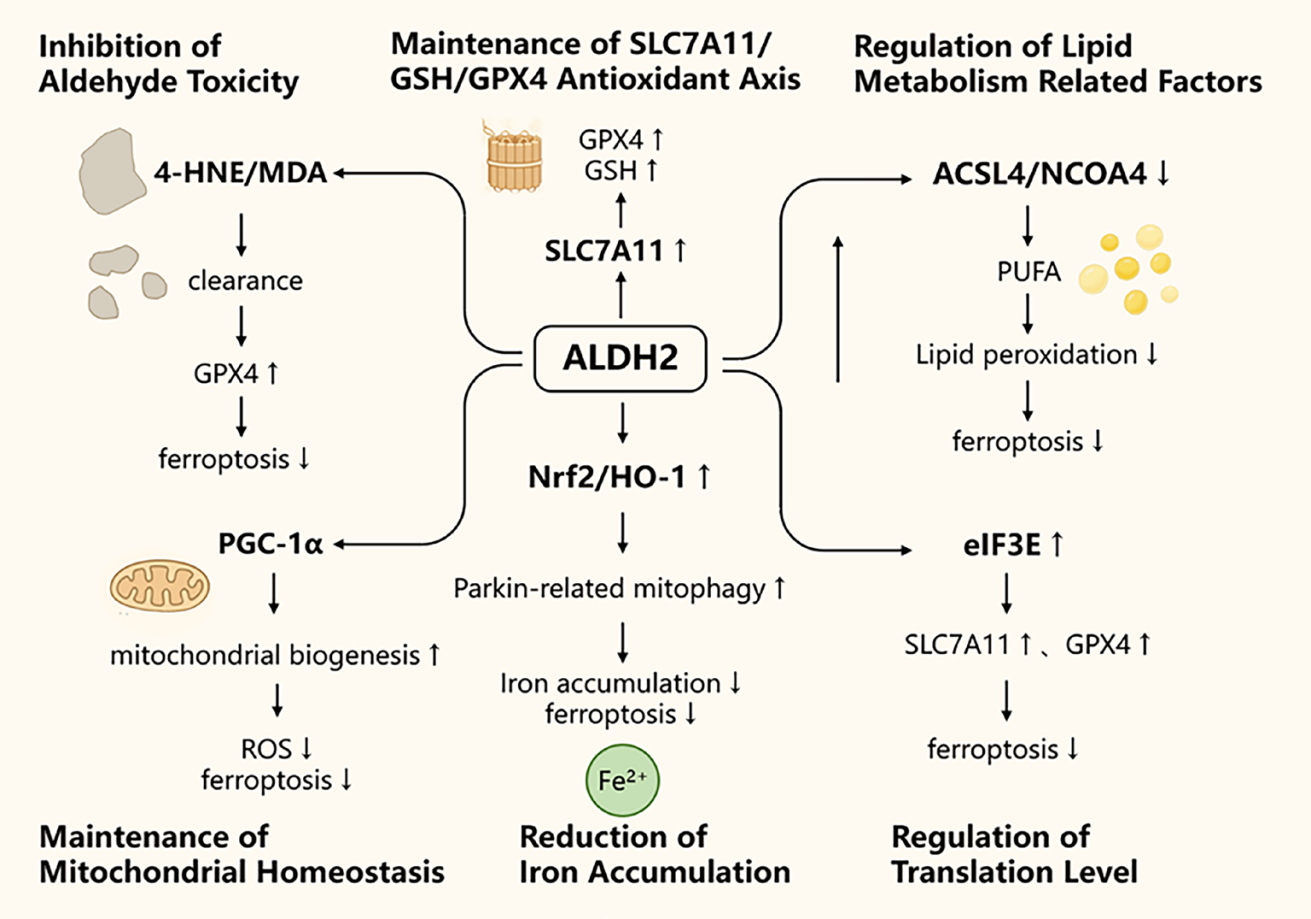
Major pathways by which ALDH2 regulates ferroptosis. ALDH2 detoxifies lipid peroxidation by‐products, particularly 4‐HNE, thereby preventing GPX4 carbonylation and preserving its stability and antioxidant activity. ALDH2 enhances SLC7A11 expression and GSH synthesis, ensuring adequate substrate supply for GPX4. By downregulating ACSL4, ALDH2 prevents polyunsaturated fatty acid (PUFA) peroxidation and alleviates ferroptosis. ALDH2 promotes PGC‐1α mediated mitochondrial biogenesis, reducing ROS production. Through Nrf2/HO‐1 signalling and Parkin‐dependent mitophagy, ALDH2 lowers free iron levels, indirectly modulating ferroptosis. Moreover, ALDH2 interacts with translational machinery components such as eIF3E, regulating the expression of SLC7A11, GPX4 and other iron metabolism‐related factors.

Ferroptosis is a form of regulated cell death driven by iron‐dependent lipid peroxidation, with GPX4 serving as the central executioner. Upstream determinants including the glutamate/cystine antiporter system Xc^−^ (SLC7A11)/GSH axis, fatty acid metabolism and mitochondrial function, collectively shape cellular susceptibility to ferroptosis [27]. ALDH2, a key mitochondrial enzyme that detoxifies reactive lipid peroxidation byproducts (most notably ROS and 4‐HNE), mitigates protein carbonylation and membrane lipid injury. These aldehydes are hallmark metabolites of lipid peroxidation that amplify oxidative stress and promote ferroptosis [59]. ALDH2 exhibits potent anti‐inflammatory, antioxidant and anti‐lipid peroxidation properties, all of which intersect with the process of ferroptosis during IRI. Thus, the functional status of ALDH2 directly dictates cellular vulnerability to ferroptosis [55, 60] The protective effect of ALDH2 in IRI is partly mediated through the inhibition of ferroptosis. Numerous reviews and experimental studies have attributed the link between ALDH2 activity and IRI outcomes to its ability to scavenge toxic aldehydes, preserve mitochondrial integrity and sustain antioxidant defences [11, 61]. Moreover, some studies have directly or indirectly assessed ferroptosis markers (such as ACSL4 and GPX4 expression, as well as lipid peroxidation products) and found that modulation of ALDH2 alters these indices. These findings support the biological and therapeutic relevance of ALDH2‐mediated anti‐ferroptotic effects in IRI [40, 62]. Building on these biochemical functions, recent studies have increasingly revealed that ALDH2 can modulate the initiation and execution of ferroptosis through multiple pathways, thereby influencing IRI in the heart, kidney and brain, as well as shaping tumour cell sensitivity to anticancer therapies [40, 63, 64]. At the molecular level, the interplay between ALDH2 and ferroptosis is primarily reflected in the following mechanisms (Figure 3).

### 3.1 Inhibition of Lipid Peroxidation and Reactive Aldehyde Accumulation

During ischemia–reperfusion, lipid peroxidation products such as 4‐HNE and MDA serve as hallmarks of ferroptosis. Among them, 4‐HNE is particularly critical and its clearance, along with the reduction of lipid peroxidation stress, represents the primary mechanism by which ALDH2 suppresses ferroptosis. 4‐HNE generated during lipid peroxidation can form alkylation adducts with proteins, damaging membrane proteins and antioxidant systems, thereby reinforcing the positive feedback loop of lipid peroxidation and impairing GPX4 function [65]. Specifically, 4‐HNE carbonylates the cysteine residue at C93 of GPX4 and C247 of OTUD5, disrupting their interaction and exacerbating GPX4 degradation through K48‐linked polyubiquitination. Activation of ALDH2 can alleviate this effect, thereby preserving GPX4 function and suppressing ferroptosis [40]. Enhancing ALDH2 activity (for instance, with the small‐molecule agonist Alda‐1) reduces 4‐HNE levels and protein adduct formation, alleviates mitochondrial dysfunction and oxidative stress and consequently lowers the likelihood of ferroptosis [66]. Evidence from both animal and in vitro models demonstrates that Alda‐1 mitigates IRI‐associated tissue injury [66], providing functional evidence that suppression of ferroptosis via reduced lipid peroxidation underlies its protective effects.

### 3.2 Maintaining the Antioxidant System (SLC7A11/GSH/GPX4 Axis)

ALDH2 influences the upregulation of cellular antioxidant pathways, thereby stabilising GPX4 function. Recent in vivo and in vitro studies indicate that ALDH2 activation not only reduces direct aldehyde toxicity but also promotes the maintenance or upregulation of the SLC7A11/GSH pathway, indirectly enhancing GPX4 substrate availability and activity (the ‘ALDH‐SLC7A11/GSH‐GPX4’ axis) [67]. Experimental evidence in kidney and other organ models supports this cascade, showing that ALDH2 activation suppresses ferroptosis via this pathway and improves pathological outcomes [67] ACSL4, a key enzyme in ferroptosis‐associated lipid peroxidation, is also regulated by ALDH2. In an Alzheimer's disease mouse model, ALDH2 suppresses SP1–ACSL4–mediated lipid peroxidation and ferroptosis by downregulating GPX4 and SLC7A11 while upregulating NCOA4, thereby rescuing cardiac abnormalities induced by APP/PS1 mutations [62].

### 3.3 Regulation of Mitochondrial Homeostasis

The coupling between ALDH2 and mitochondrial homeostasis/biogenesis may alter cellular sensitivity to ferroptosis [7, 68]. Mitochondria serve as a central hub for iron metabolism, ROS generation and lipid metabolism. By preserving mitochondrial function and promoting PGC‐1α–mediated mitochondrial biogenesis, ALDH2 reduces mitochondrial ROS production and modulates phospholipid metabolism, thereby lowering the threshold for membrane lipid peroxidation and ferroptosis initiation [7]. This protective role has been validated in acute kidney injury and other IRI models, underscoring the complementary relationship between ALDH2‐mediated mitochondrial protection and ferroptosis suppression [7]. ALDH2 also stabilises mitochondrial morphology and function by activating the Nrf1‐FUNDC1 signalling cascade, thereby preventing cardiac dysfunction [69]. Mechanistic studies further reveal that FUNDC1 interacts with GPX4 and facilitates its recruitment to mitochondria through the translocase of the outer/inner membrane (TOM/TIM) complex, amplifying the mitochondrial protective effects of ALDH2 [63].

### 3.4 Attenuation of Iron Accumulation

Regulation of iron homeostasis may represent another mechanism by which ALDH2 modulates ferroptosis. Within cellular iron metabolism, ALDH2 reduces 4‐HNE accumulation and activates the Nrf2/HO‐1 pathway to limit intracellular iron overload. In hepatocytes, ALDH2 alleviates ferroptosis by promoting Parkin‐dependent mitophagy [70]. Moreover, ALDH2 is associated with systemic iron storage. Genetic studies demonstrate that the ALDH2 variant rs671 is closely correlated with serum ferritin levels, with male carriers of the mutant allele exhibiting reduced ferritin concentrations [71]. Recent findings suggest that ALDH2 interacts with translational machinery components such as eIF3E, reshaping the cellular translatome and altering the expression of proteins involved in antioxidant defence and membrane homeostasis. This translational‐level regulation may not only influence SLC7A11/GPX4 expression but also modulate key factors linked to lipid metabolism and iron homeostasis, thereby indirectly shaping ferroptosis susceptibility [72].

These findings highlight the multifaceted role of ALDH2 in regulating ferroptosis and its therapeutic potential in IRI. Nonetheless, it is important to note that while extensive evidence supports ALDH2‐mediated ferroptosis suppression via attenuation of lipid peroxidation and aldehyde toxicity, the precise pathways and critical nodes vary across tissues and pathological contexts. For instance, in tumours, ALDH2 deficiency may increase ferroptosis susceptibility and influence chemotherapy sensitivity [64], whereas in parenchymal organ IRI, the protective effects of ALDH2 are more pronounced [31]. The role of ALDH2 extends beyond ferroptosis, encompassing apoptosis, necroptosis and pyroptosis. In IRI, ALDH2 not only inhibits ferroptosis by mitigating lipid peroxidation and suppressing inflammatory responses but also indirectly prevents NLRP3 inflammasome activation and caspase‐1 dependent pyroptosis [18]. Thus, ALDH2 likely occupies a central position at the intersection of multiple programmed cell death pathways during IRI. Future investigations should further dissect both the molecular level (e.g., potential direct interactions between ALDH2 and GPX4, the impact of quaternary post‐translational modifications on GPX4 activity and transcriptional/translational regulation of SLC7A11 by ALDH2) and the translational level (organ‐specific models and ALDH2 genetic polymorphisms). Such efforts will enable the refinement of the ALDH2–ferroptosis regulatory network and its translation into clinically actionable therapeutic strategies.

## 4 Other Forms of Programmed Cell Death Regulated by ALDH2 in IRI

A large body of research has shown that ischemia–reperfusion injury (IRI) involves multiple forms of programmed cell death, including apoptosis, necrosis, necroptosis, autophagy and pyroptosis. These pathways participate in the pathological process alternately or concurrently, depending on the stage of injury and the tissue type. Exploring their interplay may help uncover novel mechanisms of ferroptosis regulation [1]. Recent studies demonstrate that ALDH2 activation not only alleviates ferroptosis but also exerts protective effects in apoptosis, pyroptosis and autophagy [18]. During different stages of IRI, cells undergo distinct modes of death. For example, in ischemic stroke, the mode of neuronal death varies with ischemic duration, oxygen availability and residual blood flow: under moderate damage, autophagy may serve as a protective mechanism to sustain cell viability, whereas severe injury typically manifests as irreversible necrosis, apoptosis and ferroptosis [73, 74]. Thus, ALDH2 has emerged as a central regulator of autophagy, apoptosis, necroptosis and ferroptosis during IRI [22, 75, 76]. Overall, cell death modes in IRI evolve dynamically, highlighting the temporal complexity of programmed death under ischemic conditions and underscoring ALDH2's multifaceted role in determining cell fate across pathological contexts. By modulating apoptosis, necroptosis, autophagy and ferroptosis, ALDH2 serves as a key determinant of cellular outcomes. Elucidating this complex network may offer new targets for indirect regulation of ferroptosis and promote optimization of therapeutic strategies for IRI.

### 4.1 ALDH2 and Apoptosis

Apoptosis is a classical energy‐dependent form of programmed cell death, characterised by distinctive cellular morphological changes. In the early stages of apoptosis, cells exhibit shrinkage, increased cytoplasmic density and more compactly arranged organelles under the microscope. Nuclear condensation is also observed, with chromatin condensation being a hallmark feature of apoptosis. Biochemically, apoptosis is typically manifested as the activation of a complex caspase‐dependent cascade, which is energy‐dependent [77, 78].

Mechanistically, apoptosis is mainly mediated through two classical pathways: (1) The extrinsic pathway (death receptor pathway), which involves classical ligand‐receptor interactions on the cell surface. Death receptors such as Fas/CD95, TNFR1 and TRAIL‐R1/2 recruit downstream adaptor proteins like TRADD and FADD upon ligand binding, activating initiator caspases (e.g., caspase‐8) and ultimately triggering the apoptotic cascade. (2) The intrinsic pathway (mitochondrial pathway), which is initiated by mitochondrial outer membrane permeabilization (MOMP) and loss of mitochondrial integrity. Upon increased mitochondrial permeability, cytochrome c is released into the cytoplasm, binds Apaf‐1 and activates caspase‐9, which subsequently activates effector caspase‐3. This process is finely regulated by Bcl‐2 family proteins, where the ratio of pro‐apoptotic proteins (Bax, Bak) to anti‐apoptotic proteins (Bcl‐2, Bcl‐xL) determines mitochondrial membrane stability [77, 79].

Ample evidence indicates that apoptosis is widespread in IRI and is closely associated with ROS and lipid peroxidation. ALDH2 plays an important role in apoptosis regulation by clearing reactive aldehydes and ROS [80, 81]. Studies across different organ models have revealed broad regulatory relationships between ALDH2 and cell apoptosis. In cardiac IRI models, ALDH2 reduces the accumulation of 4‐HNE and MDA through the ERK/p38 signalling pathway, thereby decreasing cardiomyocyte apoptosis [81]. In renal IRI models, ALDH2 upregulation reduces tubular cell apoptosis via the IκBα/NF‐κB/IL‐17C pathway [21]. In hepatic IRI models, Alda‐1 activation of ALDH2 promotes autophagy through AKT/mTOR and AMPK pathways, indirectly suppressing hepatocyte apoptosis [82]. In the nervous system, ALDH2 activation similarly exhibits marked anti‐apoptotic effects [18]. Within the intrinsic apoptotic pathway, ALDH2 regulates the Bax/Bcl‐2 ratio through upstream AKT signalling, thereby stabilising mitochondrial function and reducing caspase‐3 activation [83]. For instance, in porcine myocardial IRI models, increased ALDH2 expression correlates with a higher Bcl‐2/Bax ratio and decreased caspase‐3 activity, associated with improved cardiac functional recovery [84]. Clinical sample studies also demonstrate that high ALDH2 expression stabilises mitochondrial membrane potential in patient cardiomyocytes and significantly lowers caspase‐3 activation via the PI3K/AKT/mTOR pathway [11].

Apoptosis and ferroptosis share certain molecular cross‐talk. Studies have shown that pro‐apoptotic BCL‐2‐specific BH3 mimetics increase sensitivity to GPX4 inhibition‐induced ferroptosis, leading to MOMP and holocytochrome c release [85]. In diffuse large B‐cell lymphoma cells dependent on NF‐κB and STAT3 survival signalling, DMF treatment effectively induces ferroptosis while inhibiting IKK complex and JAK kinase activity, suppressing NF‐κB/STAT3 signalling [86]. This indicates that apoptosis‐related molecules not only determine the occurrence of apoptosis but may also influence susceptibility to ferroptosis by modulating ROS levels and mitochondrial membrane homeostasis. ALDH2 significantly inhibits cell apoptosis in the context of IRI by clearing harmful aldehydes, regulating the Bcl‐2 family balance and activating PI3K/AKT/mTOR signalling. Meanwhile, apoptotic signals intersect with ferroptosis at multiple molecular nodes, suggesting that ALDH2's role in coordinating these two forms of cell death may have broader mechanistic implications.

### 4.2 ALDH2 and Necroptosis

Necroptosis is a regulated form of programmed necrosis triggered by non‐physiological stimuli such as physical, mechanical, or chemical stressors [87]. Its morphological features resemble traditional necrosis, including cell swelling, membrane rupture and cytoplasmic content leakage, but at the molecular level, it is highly controlled. Necroptosis is mediated by a signalling cascade involving receptor‐interacting protein kinase 1 (RIPK1), RIPK3 and mixed lineage kinase domain‐like protein (MLKL) and is considered a prototypical ‘programmed necrosis’ [87]. In the context of IRI, necroptosis commonly occurs in the mid‐to‐late reperfusion phase and is regarded as a critical mechanism exacerbating inflammation and the extent of myocardial infarction.

The classical trigger of necroptosis is the TNF‐α/TNFR1 signalling pathway. Upon TNF‐α binding to TNFR1, Complex I is initially formed, comprising TNF receptor‐associated death domain protein (TRADD), TNF receptor‐associated factor 2 (TRAF2), cellular inhibitor of apoptosis proteins (cIAP1 or cIAP2) and the linear ubiquitin chain assembly complex (LUBAC). Its primary function is to maintain cell survival signalling through K63‐linked ubiquitination of RIPK1 [88]. If Complex I is destabilised or blocked, TNF signalling shifts to form Complex IIa/IIb, which contains Fas‐associated death domain protein (FADD) and caspase‐8, thereby initiating the apoptotic pathway [89]. When caspase‐8 is inhibited, RIPK1 interacts with RIPK3 and phosphorylates MLKL, forming the necrosome, which drives plasma membrane rupture and induces necroptosis [89]. Besides the TNF pathway, death receptors such as Fas, DR3/DR4/DR5/DR6 and pattern recognition receptors like TLR3 and TLR4 can also activate necroptosis in a RIPK3‐dependent manner under caspase inhibition. Moreover, pattern recognition receptors (PRRs) such as TLR3 and TLR4 promote necrosome formation via TRIF, IFN‐β, RIPK3 and MLKL in the presence of caspase inhibitors, thereby inducing necroptosis [89].

ROS accumulation and Ca^2+^ overload trigger necroptosis mediated by inflammatory factors. Conversely, key necroptotic factors such as RIPK3 can also induce inflammation and ROS generation [90, 91]. Necroptosis generally occurs in the late reperfusion phase and persists for a prolonged period [1]. Studies indicate that inhibiting RIPK3 within the first 10 min of reperfusion does not alleviate cardiac injury. Further research suggests that in the early stage of IRI, RIPK3 mainly mediates reperfusion injury via oxidative stress and mitochondrial activity rather than necroptosis [92]. Other studies have shown that necroptosis inhibitors such as Nec‐1 provide significant cell protection in the late infarction phase and can enhance long‐term organ function after IRI. However, their efficacy is minimal when administered during the early infarction phase [93, 94].

ALDH2 inhibits necroptosis mainly through its detoxification of toxic aldehydes and antioxidative function, thereby maintaining intracellular Ca^2+^ homeostasis and stabilising mitochondrial function [95]. ALDH2 exerts its protective role via several mechanisms: (1) Inhibiting caspase‐related pathways: ALDH2 downregulation is associated with abnormal caspase activation, exacerbating necroptosis, whereas normal ALDH2 activity maintains a balance between apoptosis and necroptosis [96]. (2) Downregulating the RIPK1/RIPK3/MLKL pathway: During oxidative stress, the RIPK1/RIPK3/MLKL signalling cascade is upregulated, leading to increased necroptosis. In glutamate‐induced excitotoxic retinal models of glaucoma, reduced ALDH2 expression elevates RIPK1/RIPK3/MLKL expression, enhancing necroptotic cell death [97]. In alcohol‐induced cardiac injury models, decreased ALDH2 activity increases caspase activation and RIPK1/RIPK3/MLKL‐mediated necroptosis [98]. Similarly, in high‐glucose experimental models, reduced ALDH2 expression is associated with increased ROS generation and upregulation of RIPK1, RIPK3 and MLKL, resulting in enhanced necroptosis [75, 99]. (3) Preventing 4‐HNE accumulation: ALDH2 inhibition leads to 4‐HNE accumulation. In vitro studies show that 4‐HNE promotes cardiomyocyte necroptosis by reducing RIPK1 ubiquitination and proteasomal degradation. In mouse models, 4‐HNE perfusion exacerbates necroptosis in a time‐ and concentration‐dependent manner [100].

Recent studies reveal that necroptosis and ferroptosis are not entirely independent but are signal‐coupled. In Parkinson's disease models, mitochondrial dysfunction inhibits caspase‐8 activation, shifting apoptotic signalling toward the RIPK1/RIPK3/MLKL pathway and further triggering ferroptosis under high ROS conditions [101]. Other research shows that TNF‐α signalling can regulate glutathione (GSH) synthesis to protect synovial cells from ferroptosis, suggesting a dual role of the TNF‐α–RIPK pathway in ferroptosis susceptibility [102]. This necroptosis–ferroptosis amplification effect often produces mixed or sequential cell death phenotypes in IRI, particularly in myocardial infarction and cerebral ischemia models.

In summary, necroptosis plays a crucial role in the mid‐to‐late phase of IRI, depending on TNF‐α–RIPK1/RIPK3/MLKL signalling and ROS amplification. ALDH2 plays a key inhibitory role by suppressing caspase activity, downregulating RIPK1/RIPK3/MLKL and preventing 4‐HNE accumulation. Additionally, the crosstalk between necroptosis and ferroptosis further indicates that ALDH2 is not only an inhibitor of necroptosis but also a potential indirect regulator of ferroptosis, making it an attractive therapeutic target for future IRI interventions.

### 4.3 ALDH2 and Pyroptosis

Pyroptosis is a canonical inflammatory form of programmed cell death. Compared with apoptosis, it does not preserve plasma membrane integrity but is characterised by rapid cell swelling, membrane rupture and the eventual release of pro‐inflammatory cytokines such as IL‐1β and IL‐18 [103], leading to a strong inflammatory response. The hallmark of this process is the cleavage of Gasdermin D (GSDMD) by inflammatory caspases (caspase‐1/4/5/11), producing an N‐terminal fragment that forms pores in the plasma membrane, causing cellular contents such as IL‐1β and IL‐18 to be released, thereby exacerbating local and systemic inflammation under pathological conditions like IRI [103].

Pyroptosis is usually initiated by inflammasome assembly. Triggered by pathogen‐associated molecular patterns (PAMPs) or damage‐associated molecular patterns (DAMPs), receptors such as NLRP3, AIM2 and NLRC4 sense these signals and recruit the adaptor protein apoptosis‐associated speck‐like protein containing a CARD (ASC) to activate caspase‐1. This leads to GSDMD cleavage and the release of pro‐inflammatory cytokines, forming the canonical inflammatory pathway [104]. Activated caspase‐1 not only cleaves GSDMD to induce pore formation but also matures pro‐IL‐1β and pro‐IL‐18, triggering an immune response [104]. Additionally, a non‐canonical pyroptosis pathway exists: in mice, it is mediated by caspase‐11, while in humans, caspase‐4/5 directly recognises cytosolic lipopolysaccharide (LPS) without inflammasome assembly, cleaving GSDMD and inducing pyroptosis [105, 106]. In myocardial IRI, pyroptosis has been identified as a key mode of cell death, with oxidative stress, calcium overload and inflammatory cascades collectively contributing to cell death, significantly impacting myocardial injury and functional recovery during IRI [107]. Studies show that pro‐inflammatory cytokine levels rise sharply in the early reperfusion phase and ROS along with calcium overload drive the activation of the NLRP3–caspase‐1–GSDMD pathway, leading to pyroptosis and promoting cardiomyocyte necrosis, uncontrolled inflammation and fibrotic remodelling [108].

As an aldehyde detoxifying enzyme, ALDH2 can indirectly inhibit inflammasome activation by reducing ROS and toxic aldehyde levels, thereby mitigating pyroptotic responses. Under IRI conditions, excessive ROS can activate the NLRP3 inflammasome and induce pro‐caspase‐1 cleavage into active caspase‐1. Active caspase‐1 further cleaves GSDMD, whose N‐terminal fragment inserts into the plasma membrane to form pores, resulting in the release of pro‐inflammatory cytokines (mature IL‐1β and IL‐18) and mediating pyroptosis [109]. Studies have shown that ALDH2 negatively regulates inflammasome activation by inhibiting the ROS–NLRP3 pathway, thereby reducing GSDMD cleavage and IL‐1β release and alleviating tissue damage [20]. In mouse models of myocardial IRI, the ALDH2 activator Alda‐1 decreases caspase‐1 activity and GSDMD‐NT levels and reduces the release of inflammatory cytokines, exhibiting significant cardioprotective effects [110]. Conversely, ALDH2 deficiency exacerbates pyroptosis in myocardial IRI. In ALDH2 knockout mice, NLRP3 inflammasome activation is enhanced, caspase‐1 cleavage is increased and GSDMD pore formation rises, leading to elevated IL‐1β release and myocardial injury [110]. The ALDH2 activator Alda‐1 significantly suppresses oxidative stress–mediated activation of the NLRP3 inflammasome, reduces Gasdermin D expression, thereby alleviating pyroptosis‐associated cardiac and neurological injury and improving post‐resuscitation cardiac and neurological function. Mechanistically, ALDH2 blocks NLRP3/ASC complex assembly by degrading 4‐HNE [111].

Pyroptosis and ferroptosis intersect and amplify each other at multiple molecular levels: in melanoma cells, iron induces mitochondrial outer membrane protein Tom20 oxidation and oligomerization via ROS, recruiting Bax to promote cytochrome c release, activating caspase‐3 and cleaving GSDME, ultimately triggering GSDME‐dependent pyroptosis. This suggests that iron‐activated ROS can induce pyroptosis via the Tom20/Bax/caspase/GSDME pathway [112]. Strong pyroptotic responses accompanied by ROS and Ca^2+^ imbalance can promote lipid peroxidation and iron release, reducing cellular resistance to ferroptosis. Conversely, inhibition of the pyroptotic pathway (e.g., blocking the NLRP3/caspase‐1/GSDMD axis) can indirectly mitigate ferroptosis [113].

### 4.4 Identifying Ferroptosis Regulatory Nodes From Other Programmed Cell Deaths

In the pathological context of ischemia–reperfusion injury (IRI), which combines high metabolic stress with oxidative damage, cells rarely undergo a single mode of regulated cell death (RCD). Instead, multiple death pathways often occur in parallel, intersect and sequentially amplify each other. Various forms of regulated cell death do not occur in isolation but are interconnected through shared upstream signals, such as mitochondrial dysfunction, reactive oxygen species (ROS), lipid peroxidation and iron metabolism dysregulation, ultimately determining the type and extent of tissue injury [1]. Identifying and targeting key nodes that control ferroptosis from other RCD pathways helps both to elucidate the complex mechanisms of IRI and to provide novel entry points for therapeutic intervention. Mitochondria serve as both the central hub for energy metabolism and a signalling center for cell death. Ischemia leads to energy depletion, Ca^2+^ overload and electron transport chain dysfunction, while reperfusion causes extensive electron leakage and ROS production. ROS, together with lipid peroxidation products such as 4‐hydroxynonenal (4‐HNE), act on membranes, proteins and nucleic acids, representing a shared upstream trigger for multiple death pathways. These shared upstream signals can directly compromise membrane integrity or modulate death pathways by influencing inflammasome activation or caspase signalling [114]. The various forms of regulated cell death involved in IRI are summarised in Table 2.

**TABLE 2:** The role of different programmed cell death in IRI.

RCD	Molecular mechanism	Functional characteristics in IRI	Regulatory effect of ALDH2	Crosstalk with ferroptosis
Apoptosis	Exogenous pathways: Fas/CD95, TNFR1 → caspase‐8 activation Endogenous pathways: Imbalance of Bax/Bcl‐2 ratio→mitochondrial outer membrane permeability↑ → cytochrome c release→Apaf‐1/caspase‐9 → caspase‐3 [77, 79].	It is active in the early stage of IRI and widely exists in organs such as the heart, brain, kidneys and liver. It is closely related to ROS and lipid peroxidation [21, 81, 82, 83].	Clear 4‐HNE and MDA, regulate the ratio of Bcl‐2/Bax, stabilise mitochondria and inhibit caspase activation; Activate the PI3K/AKT/mTOR pathway [11, 81, 83].	The Bax/Bcl‐2 ratio, mitochondrial ROS and Bcl‐2‐specific BH3 mimics can affect ferroptosis sensitivity [85, 86].
Necroptosis	TNF‐α/TNFR1 → ubiquitination of RIPK1 → phosphorylation of MLKL→necrosome formation→cell membrane rupture [88, 89].	It often occurs in the middle and late stages of reperfusion, aggravating the inflammatory response and the extent of infarction [1, 92].	Degrade 4‐HNE and maintain the steady state of Ca^2+^; Down‐regulate the RIPK1/RIPK3/MLKL pathway; Reduce the accumulation of ROS and inhibit necrotic apoptosis [96, 97, 100].	The RIPK1/RIPK3/MLKL pathway induces ROS production amplification and oxidative stress. TNF‐α signalling is tunable for GSH synthesis [101, 102].
Pyroptosis	Assembly of NLRP3/AIM2/NLRC4 inflammasome→activation of caspase‐1 → cleavage of GSDMD→formation of membrane pores, release of IL‐1β and IL‐18 [104].	Inflammatory factors increase in the early stage of reperfusion. The imbalance between ROS and Ca^2+^ drives pyroptosis, promoting myocardial injury and fibrosis [107, 108].	Clear ROS/4‐HNE and block NLRP3/ASC assembly; Reduce the activation of caspase‐1 and GSDMD and the release of IL‐1β; ALDH2 alleviates myocardial and brain injuries [19, 20, 109, 110].	Iron‐induced ROS promotes the Tom20/Bax/caspase‐3/GSDME pathway; Pyroptosis can promote lipid peroxidation and iron release, aggravating ferroptosis [112, 113].

#### 4.4.1 Crosstalk Between Apoptosis and Ferroptosis

The apoptotic pathways, particularly the intrinsic mitochondrial pathway, regulate mitochondrial membrane permeability, cytochrome c release and caspase activity. These processes not only determine the initiation of apoptosis but also significantly alter redox balance and lipid metabolism, thereby modulating the lipid peroxidation threshold and glutathione peroxidase 4 (GPX4) function [115]. Changes in the ratio of pro‐apoptotic Bax to anti‐apoptotic Bcl‐2 influence apoptosis susceptibility and indirectly modulate ferroptosis sensitivity through mitochondrial ROS [116]. Furthermore, BH3 mimetics, which mimic the BH3 domain of Bcl‐2 family proteins, have been reported to enhance ferroptosis induced by GPX4 inhibition [117], indicating that apoptotic factors can act as upstream amplifiers of ferroptosis. Therefore, targeting Bcl‐2 family regulation, preserving mitochondrial integrity, or inhibiting pro‐apoptotic signals represents a critical approach to restraining ferroptosis from the perspective of apoptosis [85].

#### 4.4.2 Cross‐Talk Between Necroptosis and Ferroptosis

Necroptosis, driven by the RIPK1/RIPK3/MLKL signalling axis, can promote ROS accumulation and mitochondrial dysfunction, thereby further enhancing lipid peroxidation. Activation of RIPK3 during necroptosis alters mitochondrial function and ROS production and prolonged or intense necroptosis facilitates the accumulation of lipid peroxides, eventually triggering ferroptosis [68]. In some disease models, necroptosis inhibition (e.g., with Nec‐1) provides late‐stage protection but is less effective early, suggesting that early ROS/mitochondria‐mediated, non‐MLKL mechanisms—favourable for ferroptosis—may dominate initial injury [118]. Combining RIPK signal modulation with lipid peroxide clearance may offer a novel therapeutic strategy for co‐targeting necroptosis and ferroptosis.

#### 4.4.3 Cross‐Talk Between Pyroptosis and Ferroptosis

Pyroptosis induces membrane pore formation and cytokine release via the NLRP3–caspase‐1/11–gasdermin D (GSDMD) axis. Activation of inflammasomes, particularly NLRP3, leads to caspase‐1/11‐mediated GSDMD cleavage, pore formation and release of IL‐1β/IL‐18 [68]. This strong inflammatory response and membrane damage exacerbate ROS accumulation, iron release and intracellular Ca^2+^ imbalance, indirectly promoting lipid peroxidation and compromising cellular antioxidant capacity against ferroptosis [113]. Blocking the NLRP3–caspase–GSDMD pathway not only reduces inflammation but also mitigates ferroptosis induced by aldehyde toxicity [113]. Furthermore, NLRP3 has been shown to synergize with ferroptosis inhibitors, such as ferrostatin‐1, in protecting tissues during IRI [119], highlighting inflammasome/pyroptosis nodes as effective targets to suppress ferroptosis.

#### 4.4.4 Ferroptosis as a Central Executor

Compared with the above RCD modalities, ferroptosis is more dependent on iron metabolism and membrane phospholipid peroxidation. Its hallmark features include iron‐dependent lipid peroxidation, GPX4 inactivation, glutathione (GSH) depletion and accumulation of oxidised polyunsaturated phospholipids (PUFA‐PLs) [59]. In myocardial IRI models, substantial evidence shows that ferroptosis contributes to cardiomyocyte death at both early and delayed phases. For instance, Alox15‐induced 15‐HpETE acts as a trigger promoting cardiomyocyte ferroptosis and inhibition of ALOX15 or restoration of GPX4 attenuates IRI damage [28]. Moreover, ferroptosis exhibits bidirectional amplification with other RCD pathways. Mitochondrial damage alters lipidomic profiles and increases phospholipid oxidation, heightening ferroptosis sensitivity [120]. Conversely, ferroptotic lipid peroxidation can trigger inflammatory signals or sensitise cells to necroptosis or pyroptosis by disrupting membranes and mitochondria [121]. Iron‐dependent lipid peroxidation and dysregulated iron homeostasis thus interconnect multiple RCD pathways. On one hand, ferroptosis, driven by GPX4 inactivation, PUFA oxidation and free iron, directly destabilises membranes and compromises mitochondrial function while amplifying ROS and lipid peroxidation to facilitate inflammasome and necroptotic signalling [29]. ROS activated by iron can induce gasdermin‐mediated membrane rupture through the Tom20/Bax/caspase/GSDME axis, creating molecular cross‐talk between ferroptosis and pyroptosis [112]. On the other hand, RIPK1/RIPK3 activation amplifies ROS and inflammatory factors, further promoting ferroptotic lipid peroxidation [122]. Inhibition of ferroptosis simultaneously reduces oxidative stress and inflammation amplification, thereby mitigating IRI at the systemic level [114]. Intense lipid peroxidation may also facilitate MLKL‐mediated membrane rupture, producing hybrid or sequential cell death phenotypes at the cellular level [123]. Overall, cellular outcomes in IRI result from the ‘collusion’ and ‘competition’ of multiple RCD pathways rather than a single death modality. Ferroptosis, as a key executor, not only directly induces membrane phospholipid peroxidation and mitochondrial collapse but also amplifies ROS, iron dysregulation and inflammatory signalling to promote apoptosis, necroptosis and pyroptosis. Inhibiting ferroptosis can reduce systemic oxidative stress and inflammation, thereby protecting tissues from IRI. This cross‐talk underscores the importance of identifying and intervening in ferroptosis regulatory nodes from other RCD pathways as a promising direction for developing IRI therapies.

## 5 Ferroptosis‐Related Molecules in Downstream Pathways of ALDH2

Within the complex pathological context of IRI, ferroptosis, a form of regulated cell death dependent on iron and lipid peroxidation, has been recognised as a central driver of tissue damage. ALDH2 functions not only as a detoxifying enzyme but also as a critical regulator across multiple downstream signalling pathways. By metabolising reactive aldehydes, scavenging reactive ROS and preserving mitochondrial homeostasis, ALDH2 can directly or indirectly modulate the onset and progression of ferroptosis. Thus, identifying ferroptosis‐associated molecular nodes downstream of ALDH2 not only deepens mechanistic insights into IRI but also opens new avenues for therapeutic intervention.

### 5.1 ROS

Aberrant accumulation of ROS is a hallmark of ferroptosis. ROS comprise highly reactive oxygen‐derived molecules, including superoxide anion (O_2_•^−^), hydrogen peroxide (H_2_O_2_) and hydroxyl radicals (•OH), which are normally generated as metabolic byproducts [124]. Under pathological stress, their excessive buildup drives oxidative damage to proteins, lipids and DNA, ultimately leading to cellular dysfunction and death. ALDH2 not only detoxifies ROS directly [3] but also supports mitochondrial oxidative phosphorylation by catalysing acetaldehyde metabolism into acetate, accompanied by the conversion of NAD^+^ to NADH, thereby sustaining the activity of respiratory complex I and indirectly limiting ROS generation [125]. Moreover, ROS levels reciprocally regulate ALDH2 activity and stability, forming a feedback loop [126, 127].

In studies investigating the potential mechanisms of IRI, evidence has further highlighted the role of ALDH2 in reducing ROS production and protecting tissues and organs. Recent research offers notable insights into this phenomenon: in diabetic myocardial IRI rat models and H9C2 hypoxia/reoxygenation cardiomyocyte experiments, Tan et al. [11] observed that upregulation of ALDH2 decreased mitochondrial ROS levels and improved the balance between mitochondrial fusion and fission, thereby maintaining cellular morphology and function. Furthermore, Xu et al. [22] reported an intriguing finding regarding ALDH2 activation: activation of ALDH2 could enhance autophagic activity by promoting phosphorylation of Beclin‐1 at the Ser90 site. This autophagic activation helps maintain cellular and tissue homeostasis by reducing ROS levels and facilitating the clearance of damaged organelles.

Collectively, these findings emphasise the multifaceted protective mechanisms coordinated by ALDH2 under IRI conditions. ALDH2 exerts a dual role in regulating ROS levels: it acts both as a direct scavenger and as a regulator through mitochondrial metabolism and autophagic pathways to maintain cellular homeostasis. These insights significantly advance our understanding of the complex interplay between ALDH2 and IRI and provide a solid foundation for its role in suppressing ferroptosis.

### 5.2 Lipid Peroxidation

Lipid peroxidation represents another hallmark of ferroptosis. Elevated intracellular ROS can directly attack lipids, with membrane lipids being among the most vulnerable targets to ROS‐induced oxidative stress. During IRI, uncontrolled oxidative stress disrupts the delicate balance between ROS production and clearance, leading to intracellular accumulation of endogenous ROS [128]. Among the ROS species involved, •OH and hydroperoxyl radicals (HO_2_•) are the main culprits. Cell membranes are rich in polyunsaturated lipids, which are directly attacked by these radicals at the carbon–carbon double bonds of polyunsaturated phospholipids, initiating chain oxidation cascades. This results in compromised membrane integrity and function [129, 130, 131].

During ischemia–reperfusion, mitochondria are the primary sites of ROS production. Accumulated ROS can directly attack mitochondrial membrane phospholipids enriched in PUFAs, triggering chain reactions that generate primary products, lipid hydroperoxides (LOOH). Secondary reactions yield over 200 highly reactive lipid‐derived aldehydes (LDAs), including 4‐HNE, MDA, 4‐oxo‐2‐nonenal, acrolein, crotonaldehyde and propionaldehyde [132]. These LDAs are not only downstream products of ROS but also further promote mitochondrial ROS generation, amplifying oxidative stress‐induced tissue damage via positive feedback. Excessive reactive aldehydes and ROS diffuse beyond mitochondria, permeating the entire cell, leading to protein dysfunction, DNA damage, gene mutations and ultimately contributing to diseases such as cardiovascular disorders, cancer, diabetes, osteoporosis and neurodegeneration.

As products of lipid peroxidation, 4‐HNE and MDA are considered biomarkers of oxidative stress and are identified as the most reactive aldehydes [133]. ALDH2, an enzyme responsible for metabolising these compounds, converts MDA into malonic acid (MOA) or acetaldehyde and 4‐HNE into 4‐hydroxy‐2‐nonenoic acid (NHA) [134].

### 5.3 4‐HNE

As previously described, 4‐HNE is a result of lipid peroxidation. ω‐6 PUFAs (such as linoleic acid, γ‐linolenic acid, or AA) produce 4‐HNE under ROS‐mediated oxidation [135]. Notably, 4‐HNE targets redox‐related signalling molecules or upstream regulatory molecules, including thioredoxin (an intracellular ROS scavenger) and GSH [136]. Moreover, 4‐HNE significantly impacts gene expression regulation and post‐translational modifications of proteins. It upregulates transcription factors such as NF‐κB and AP‐1 and can modify antioxidant enzymes like GPX4, leading to their inactivation and diminished anti‐lipid peroxidation capacity [137, 138]. Additionally, 4‐HNE participates in activating the transcription factor Nrf2, which in turn trans‐activates antioxidant response elements, upregulating genes crucial for cellular antioxidant defence and regulation of oxidative stress [139].

4‐HNE is closely associated with a range of oxidative stress‐related diseases, including neurodegenerative disorders, macular degeneration, cardiovascular diseases, atherosclerosis, metabolic syndrome and cancer. Under these pathological conditions, 4‐HNE exceeds its role as a mere marker of oxidative stress, functioning as a critical pathogenic factor [139]. Elevation of 4‐HNE induced by lipid dysregulation triggers ferroptosis in murine chondrocytes, contributing to the development of osteoarthritis in mice [140]. Reducing levels of 4‐HNE via Ferrostatin‐1 alleviates ferroptosis in LPS‐induced acute lung injury [141]. Additionally, isorhapontigenin can suppress excessive ROS production and lower 4‐HNE and 8‐OHdG levels by activating the PKCε/Nrf2/HO‐1 pathway, thereby significantly mitigating oxidative stress damage induced by cerebral ischemia–reperfusion [142]. Similar protective effects have been observed in intestinal IRI, where the IRI protector corilagin significantly attenuates oxidative stress and lipid peroxidation (e.g., 4‐HNE accumulation) and maintains iron homeostasis by inhibiting NCOA4‐dependent ferritinophagy, reducing ferroptosis [143].

In cellular environments, 4‐HNE can react with various components to form adducts, causing tissue damage [137]. ALDH2 protects against IRI‐induced damage by efficiently clearing 4‐HNE [134]. ALDH2 plays a key role in inactivating 4‐HNE; loss of ALDH2 exacerbates iron‐overload‐induced ferroptosis in cardiomyocytes, whereas ALDH2 activation alleviates ferroptosis [40]. Impaired ALDH2 activity leads to mitochondrial aldehyde accumulation and elevated oxidative stress, accompanied by increased glutathione synthesis and metabolic remodelling, showing compromised tolerance to acute oxidative stress induced by ischemia/reperfusion [144]. Moreover, studies in diabetic myocardial IRI models indicate that ALDH2 activation not only improves cardiac hemodynamics and reduces myocardial injury but also inhibits mitochondrial permeability transition pore (mitoPTP) opening by decreasing 4‐HNE accumulation, thus maintaining mitochondrial homeostasis [11]. In hepatic IRI models, the ALDH2 activator Alda‐1 significantly reduces 4‐HNE accumulation and oxidative stress, reverses mitochondrial structural damage, inhibits hepatocyte apoptosis and attenuates inflammation, overall improving liver function [82]. These findings collectively demonstrate that ALDH2 alleviates oxidative stress and ferroptosis by clearing 4‐HNE, exerting key protective effects across various tissues and organs during IRI.

### 5.4 MDA

MDA is a typical degradation product of PUFAs, particularly from AA and docosahexaenoic acid. PUFAs with more than two methylene‐interrupted double bonds are especially sensitive to oxidative damage, making them prone to MDA formation [145]. MDA detection was initially developed in food chemistry, using thiobarbituric acid (TBA) colorimetric or fluorescence methods to assess rancidity of ω‐3 and ω‐6 fatty acids [146]. This method was later applied in biomedical research but has been criticised for significant non‐specificity. Compounds in biological samples that react similarly to TBA‐MDA complexes can lead to overestimation of MDA levels. Additionally, MDA or MDA‐like substances can be generated from various precursors in lipid peroxidation chain reactions, including oxidised lipids, 2‐alkenals and 2,4‐alkadienals [145].

Although 4‐HNE has been extensively studied as a toxic signalling molecule of lipid peroxidation, its production accounts for only about 10% of that of MDA [147]. In contrast, the role of MDA as a signalling molecule or regulator of gene expression has been relatively less investigated. Nevertheless, MDA has attracted attention as a biomarker in various diseases, including cancer, cardiovascular diseases and neurodegenerative disorders, with its levels in patient blood, urine and exhaled breath condensate used for disease detection [148]. MDA‐modified low‐density lipoprotein (LDL) particles play a critical role in the pathogenesis of atherosclerosis. These modified LDL particles are taken up by vascular wall macrophages via scavenger receptors, forming lipid‐laden foam cells that constitute the core drivers of early atherosclerotic lesions. Meanwhile, mitochondria play a central role in iron–sulfur cluster and heme biosynthesis as well as energy supply, making them vital for cellular iron homeostasis. Mitochondrial dysfunction not only disrupts iron homeostasis but also exacerbates oxidative stress and lipid peroxidation, thereby promoting MDA‐LDL deposition and the progression of atherosclerosis [149]. Additionally, MDA generated under oxidative stress can covalently modify endogenous biomolecules to form neo‐self epitopes, potentially triggering abnormal biological responses. The innate immune system recognises and neutralises these MDA epitopes through both cellular and soluble effectors to maintain homeostasis. Studies indicate that the immune effects of different MDA epitopes are mediated by protein kinase C and involve PI3K, SrcK, PLC/IP3, Erk1/2, Syk and NF‐κB signalling pathways [150].

MDA is primarily degraded through enzymatic pathways, with the ALDH family playing a central role. MDA is first oxidised to acetaldehyde by ALDH2, then further metabolised to acetic acid, which eventually enters energy metabolism and is converted to carbon dioxide and water [151]. Alternatively, MDA metabolism can occur via GSH‐dependent reactions: phosphoglucose isomerase uses GSH as a cofactor to convert MDA to pyruvaldehyde in the cytosol, which is then further metabolised to D‐lactate via the methylglyoxal enzymatic system [152]. During IRI, oxidative stress is significantly enhanced, leading to excessive MDA production and resulting in tissue dysfunction. Numerous studies indicate that reducing MDA levels can effectively mitigate IRI. In cerebral IRI models, reductions in MDA and inflammatory mediators alleviate brain tissue injury by inhibiting NF‐κB phosphorylation and nuclear translocation [153]. Similarly, in hepatic IRI, the Nrf2/SLC7A11/HO‐1 axis regulates MDA levels to suppress ferroptosis [154]. The role of MDA in the heart has also been widely studied; reducing ROS accumulation, lowering MDA levels and restoring SOD activity can mitigate oxidative stress in vitro, suppress ferroptosis and decrease apoptosis‐related protein expression, thereby protecting cells from IRI‐induced cell death [155]. Notably, MDA levels are closely associated with ALDH2 activity and studies in animal models of acute lung injury [68], cardiac [19] and hepatic [82] IRI have confirmed a negative correlation between ALDH2 activity and MDA levels.

4‐HNE and MDA are considered the most potent and extensively studied substances in IRI. ALDH2 exhibits differential sensitivity toward LPO‐derived aldehydes, with highly efficient detoxification activity, particularly for 4‐HNE, acrolein and MDA [156]. However, current research on ALDH2 and toxic aldehyde function is predominantly focused on the heart, while studies in other organs such as the brain, intestine, kidney and liver remain relatively sporadic. Structural and functional differences among tissues lead to distinct responses to oxidative stress and ALDH2 activity. For example, some studies report that oxidative stress reduces ALDH2 activity, whereas others find it remains stable [157]. Therefore, further elucidation of tissue‐specific responses of ALDH2 to oxidative stress is crucial for designing organ‐targeted IRI intervention strategies. This understanding will provide a theoretical basis for revealing tissue susceptibility differences and developing targeted therapies.

### 5.5 Mitochondria

Mitochondria serve as the primary hub for ROS production and iron metabolism in cells. ROS generated from the electron transport chain can trigger and amplify mitochondrial lipid peroxidation, thereby promoting ferroptosis [158]. Inhibition of mitochondrial lipid peroxidation or suppression of electron transport/proton pump activity can reduce ferroptosis sensitivity [159]. Mitochondria play a critical role in cellular homeostasis and abnormalities in mitochondrial morphology and function are closely linked to the development of numerous diseases. However, mitochondrial processes are highly susceptible to dysregulation. Mitochondrial fission, fusion and mitophagy are closely associated with ferroptosis [160].

ALDH2 is a mitochondrial matrix and inner membrane‐localised aldehyde dehydrogenase that oxidises acetaldehyde and lipid peroxidation‐derived reactive aldehydes (such as 4‐HNE and MDA) into corresponding acids. This process eliminates ‘harmful aldehydes’ and maintains stability of the mitochondrial respiratory chain, membrane potential and energy metabolism [161]. ALDH2 dysfunction exacerbates ROS generation, lipid peroxidation and mitochondrial impairment. Conversely, activation or upregulation of ALDH2 improves mitochondrial redox homeostasis and confers cardioprotection in contexts such as IRI, diabetes, or heart failure [162]. ALDH2 plays a central role in clearing reactive aldehydes from both endogenous and exogenous sources. Post‐translational modifications provide a complex regulatory system for enzyme activity, fine‐tuning mitochondrial detoxification pathways. Studies show that SIRT3 can alter ALDH2 lysine acetylation; during IRI, ALDH2 is inactivated by acetylation, while SIRT3‐mediated deacetylation maintains ALDH2 activity, activating the PI3K–AKT–mTOR pathway to inhibit mPTP opening, stabilise mitochondrial membrane potential and mitigate IRI [11, 163]. In diabetic cardiomyopathy, ALDH2 protects cardiac function by regulating AKT and GSK3β activation, Parkin‐mediated mitophagy and overall mitochondrial function [164]. In various cardiac diseases, ALDH2 enhances mitochondrial quality control and removal of damaged mitochondria, providing protection in metabolic cardiomyopathy (MCM), cardiac remodelling/heart failure and myocardial IRI [165].

ALDH2 inhibits ferroptosis through multiple downstream mechanisms, including regulation of ROS levels [11], clearance of lipid peroxidation‐derived reactive aldehydes [156], maintenance of GPX4 function, modulation of the SLC7A11/GSH axis, inhibition of ACSL4‐mediated lipid peroxidation pathways and enhancement of mitophagy and iron homeostasis [57]. Under pathological conditions such as IRI, ALDH2 functions not only as an antioxidant and detoxifying enzyme but also as a key regulator of ferroptosis‐related signalling [62]. Targeted activation of ALDH2 provides an important strategy for preventing and treating tissue IRI. Aldehydes such as 4‐HNE and MDA are not only markers of ferroptosis but also signalling molecules connecting other cell death pathways [166]. 4‐HNE can alkylate key proteins, promoting their inactivation or degradation, thereby increasing cellular susceptibility to ferroptosis [40]. Additionally, 4‐HNE can inhibit RIP1 ubiquitination, affect the balance between apoptosis and necroptosis and activate inflammasomes, exacerbating pyroptotic and necrotic signalling cascades [138]. Therefore, clearance or detoxification of 4‐HNE represents a critical node to interrupt these mutually reinforcing pathways. Extensive in vivo and in vitro studies have demonstrated that ALDH2 activation clears 4‐HNE, reduces ROS, stabilises mitochondrial function and downregulates the NLRP3 inflammasome, thereby simultaneously inhibiting apoptosis, necroptosis and pyroptosis while attenuating ferroptosis initiation and execution [40, 134, 161]. Specific mechanisms include: (1) degradation of 4‐HNE to protect GPX4 from aldehyde alkylation and ubiquitin‐mediated degradation, maintaining its anti‐lipid peroxidation function [40]; (2) maintaining the SLC7A11/GSH axis and increasing GPX4 substrate availability, indirectly inhibiting ferroptosis [67]; (3) suppressing ACSL4‐SP1‐mediated pro‐lipid peroxidation pathways to reduce synthesis of oxidizable phospholipids [167]; (4) promoting mitophagy and upregulating Nrf2/HO‐1 to decrease free iron accumulation and mitochondrial ROS [11, 18]. Collectively, ALDH2 functions as a key upstream node that disrupts mutually amplifying pathways, providing significant protection in IRI‐affected tissues.

## 6 Conclusion

IRI remains an important source of morbidity and mortality across cardiovascular, neurological, renal, hepatic and intestinal diseases. Despite decades of research, effective clinical strategies to mitigate reperfusion‐induced tissue injury remain limited. One of the greatest challenges in understanding IRI is the coexistence and dynamic interaction of multiple RCD pathways—including apoptosis, necroptosis, pyroptosis, autophagy‐related death and ferroptosis—which collectively shape the extent of organ dysfunction. Among these pathways, ferroptosis has recently emerged as an interesting mechanism due to its unique reliance on iron‐dependent lipid peroxidation and its capacity to amplify oxidative stress, mitochondrial damage and inflammatory cascades. The findings summarised in this review highlight ALDH2 as a critical upstream regulator within this complex network [77].

Among the numerous regulatory factors, ALDH2 is a key redox enzyme, primarily responsible for metabolising acetaldehyde and detoxifying reactive aldehydes generated from lipid peroxidation, such as 4‐HNE and MDA. Its antioxidant effects are crucial for maintaining cellular homeostasis [4]. Recent studies further reveal a significant link between ALDH2 and ferroptosis. Experiments by Qu et al. [18] demonstrated that ALDH2 activation alleviates multiple forms of cell death—including ferroptosis, apoptosis, autophagy and pyroptosis—supporting ALDH2's role in a comprehensive protective mechanism. Extensive studies have highlighted ALDH2's role in suppressing lipid peroxidation and scavenging reactive oxygen species, while also demonstrating its significant impact on multiple cell death pathways induced by ischemia–reperfusion [22, 75]. Both ALDH2 and ferroptosis represent promising therapeutic targets for mitigating IRI. Pharmacological activation of ALDH2, genetic strategies to enhance its expression or activity and targeted modulation of ferroptosis‐related pathways may represent complementary approaches for limiting reperfusion‐associated tissue damage. Further studies integrating mechanistic insights with clinically relevant models will be essential to determine how modulation of ALDH2 and ferroptosis can be translated into effective therapies for ischemia–reperfusion injury.

## Author Contributions

The authors are listed by the contributions respectively. All authors have read and agreed to the published version of the manuscript.

## Funding

The authors have nothing to report.

## Ethics Statement

The authors have nothing to report.

## Consent

The authors have nothing to report.

## Conflicts of Interest

The authors declare no conflicts of interest.

## Data Availability

Data sharing not applicable to this article as no datasets were generated or analysed during the current study.
